# Evaluation of Association between Vaginal Infections and High-Risk Human Papillomavirus Types in Female Sex Workers in Spain

**DOI:** 10.5402/2012/240190

**Published:** 2012-07-31

**Authors:** C. Rodriguez-Cerdeira, E. Sanchez-Blanco, A. Alba

**Affiliations:** ^1^Dermatology Department, CHUVI and University of Vigo, Vigo, Spain; ^2^University of Vigo, Vigo, Spain; ^3^Spanish Society of Cervical Pathology and Colposcopy, Centre for Molecular and Cellular Studies, Lugo, Spain

## Abstract

*Background*. Infection with and persistence of high-risk human papillomavirus (HR-HPV) are the strongest risk factors for cervical cancer. In addition, other genital microorganisms may also be involved in the progression of HPV-associated lesions. *Objetive*. To evaluate the association of the vaginal microbiota (*Candida* spp., *Trichomonas vaginalis*, and bacterial vaginosis) with HR-HPV infection in Spanish female sex workers (FSWs). *Methods*. This cross-sectional study involved 208 (FSWs; age, 18–49 years) who visited a sexually transmitted infection (STI) information and prevention center (SERGAS) between January 2010 and December 2011. Face-to-face interviews were carried out. Cervical and vaginal samples were examined for human papillomavirus (HPV), *Trichomonas vaginalis*, *Candida* spp., and microorganisms related to bacterial vaginosis (BV). *Results*. HR-HPV was found to be significantly associated with BV in FSWs with positive results for HPV16-related types (31, 33, 35, and 52). *T. vaginalis* was isolated in FSWs with the following HR-HPVs: 18, 45, 66, and 68. *Candida* spp. were isolated only in FSWs with HPV 18-positive infection. *Conclusion*. We demonstrate a significant prevalence of HR-HPVs in FSWs with disturbances in the vaginal microbiota.

## 1. Introduction

High-risk human papillomavirus (HR-HPV) infection is the most common sexually transmitted infection (STI) among young adult women and plays a critical role in the development of genital cancer. Recently, 15 HR oncological viral strains have been identified, including the HPV 16 group (alpha-9) of the alpha-papillomavirus genus (HPV 31, HPV33, HPV 35, HPV 52, and HPV 58) and the HPV 18 group (alpha-7; HPV39, HPV 45, HPV 59, and HPV 68) [1, 2]. Biological susceptibility to HR-HPV acquisition and reduced immune competence for clearance of HR-HPV infection could result from common treatable vaginal infections that disrupt the intricately balanced vaginal ecosystem and its innate protective mechanisms against infection and disease [3].

Earlier reports have suggested a link between genital tract inflammation and cervical cancer, only a few studies have controlled for HR-HPV genotypes, and no study has examined this infection in female sex workers (FSWs). The association between self-reported abnormal vaginal discharge and cervical intraepithelial neoplasia (CIN) in HR-HPV-infected women further suggests a link between genital tract anomalies and cervical cancer [4, 5]. Bacterial vaginosis (BV) and trichomoniasis are associated with high levels of anaerobic microorganisms and their byproducts, which can damage the vaginal epithelium, degrade cervical mucus, and cleave immunoglobulin A [6–8], and the evidence suggests an important association between HR-HPV and alterations in the vaginal microbiome [9, 10]. BV and trichomoniasis are categorized, along with vulvovaginal candidiasis, under the slight misnomer of vaginitis. *Candida albicans* is the most prevalent species in the majority of cases of asymptomatic colonization and vulvovaginal candidiasis. However, certain species of *Candida* are more pathogenic than others, induce hyphal and pseudohyphal formation, and enhance proteolytic activity and antigen modulation. These properties enable *Candida* to penetrate the mucosal surface and induce mucosal swelling, erythema, and exfoliation of cells [11]. In addition, some studies have reported that cervical cytologic abnormalities occur more often in women who have abnormal vaginal microbiota than in women without this condition. Therefore, we were interested in investigating whether women who carry *Candida* spp. are more prone to acquiring cervical cytologic abnormalities over time than women who have a known, disturbed, bacterial vaginal microbiota [9, 10].

Therefore, the purpose of the current study was to determine the prevalence of vulvovaginitis caused by *Trichomonas vaginalis*, *Candida *spp., or BV in a group of FSWs with HR-HPV infection.

## 2. Materials and Methods

### 2.1. Sample Size

Approval for the study was obtained from the Ethics Committee of Galician (Spain) Human Research. HIV-negative FSWs (age, 18–49 years) who visited an STI information and prevention center in the north-eastern region of Spain between January 2010 and December 2011 were included in the study. The sample size was calculated to allow for a prevalence of 50% or higher for HR-HPV, with a confidence interval of 95% and an error of 6.5%.

### 2.2. Statistical Analysis

The *χ* ^2^ test was used for statistical analysis, while the odds ratio (OR) was used to measure the strength of the association between vaginal infections and high-risk human papillomavirus types. Statistical tests were considered significant if the *P* value was ≤0.05. Logistic regression analysis was used to assess the simultaneous effect of more than 1 variable on the risk of HPV infection and to identify possible confounding factors. We determined the OR by using nonconditional logistic regression but using the correct definition; we could not estimate the prevalence ratio (PR) because we did not have any patients who were not exposed to the infection. The software used for the statistical analysis was the Statistical Package for the Social Sciences (SPSS), version 18.0.1.

### 2.3. Detection of HR-HPV

Cervical samples were collected with a cervical brush, and all cervical swabs were placed a 1 mL a tube containing of the Digene specimen transport medium (Digene Corporation, Gaithersburg, MD, USA) and were stored at −20°C until testing. The presence of HR-HPV infection was determined through the Digene HR-HPV test—Hybrid Capture II (HC2)—by using the HR-HPV probe B cocktail, which identified types 16, 18, 31, 33, 35, 39, 45, 51, 52, 56, 58, 59, and 68, in accordance with the manufacturer's instructions. To minimize the cost of testing, an HC2 HPV DNA test was performed for all samples with only the high-risk HPV probe mixture. Positive specimens were detected by binding of hybridization complexes onto the surface of the microplate wells coated with monoclonal antibodies specific to RNA-DNA hybrids. Immobilized hybrids were detected by the addition of an alkaline phosphatase-conjugated antibody to RNA-DNA hybrids, followed by the addition of a chemiluminescent substrate.

### 2.4. Detection of Bacterial Vaginosis

The presence of BV was evaluated microscopically in samples collected from the posterior vaginal fornix. BV was diagnosed on the basis of the presence of 3 of the following clinical and microscopic findings: vaginal pH greater than 4.5, presence of clue cells, grey homogenous vaginal discharge, and positive whiff test, in which a fishy odor is released after the addition of 10% potassium hydroxide solution to the vaginal fluid.

### 2.5. Detection of *T. vaginalis*

The vaginal swab was placed in 0.2 mL of sterile physiologic saline for wet mount evaluation and examined microscopically (400x) for motile *T. vaginalis* within 15 min. Sample collection was performed using calcium alginate swabs, which were transported in the Stuart Amies transport medium (Copan Innovation), kept at room temperature, and sent to the laboratory within 6 h of collection for culture on Roiron medium (MAIM S.L.). *T. vaginalis* was first detected by direct microscopic visualization of the fresh sample, followed by culture of the vaginal discharge on the Roiron medium and aerobic incubation at 37°C. The broth was examined microscopically for motile trichomonads on days 1, 3, and 5 of culture.

### 2.6. Detection of* Candida *spp.

Samples were collected using calcium alginate swabs and transported in the Stuart Amies transport medium. The samples were kept in a T° environment and then sent to the laboratory within 6 h, as recommended by the manufacturer. Confirmatory microbiological culture of vaginal discharge was carried out in Sabouraud chromogenic medium (*Candida* ID2 (CAN2); bioMérieux) in the case of women for whom a positive diagnosis was suspected but for whom a negative result was obtained by direct visualization. For samples that did not test positive following microbiological culture, complementary diagnosis analysis using the Vitek ID-YST system (bioMérieux) was also performed.

## 3. Results

This cross-sectional study involved FSWs who visited an STI information and prevention center in Vigo, Spain, between January 2010 and December 2011. All FSWs who visited the center were invited to participate in the study, and the refusal rate was very low.

Finally, 208 HIV-negative FSWs aged 18–49 years were involved in this study. The mean age of the study population was 27 years, whilst 60% of participants belonged to the target age group of 18–25 years. HR-HPV prevalence peaked in younger women aged 18–25 years. As expected, the prevalence of *T. vaginalis* in FSWs was high (17%). HR-HPV infection was present in 7.5–35.5% of these women, and the peak prevalence of this infection occurred 20 years earlier than that for *T. vaginalis*.

Data for HR-HPV-positive samples were grouped as belonging to HPV16-related genotypes (31, 33, 35, 52, and 58), HPV18-related genotypes (39, 45, 59, and 68), or other genotypes, including HPV51 and HPV66. In the study group of 208 FSWs, 81 women had positive results for HPV16 infection and 48 for HPV18 infection. The distribution of frequencies for different genotypes of HPV is shown in Table 1 [12].

Data from univariate analysis showed that BV was strongly associated with HR-HPV, as was the presence of *T. vaginalis*. We found no relationship between *Candida *spp. and HR-HPV, with the exception of HPV18 (Table 2).

Among women with vaginal *T. vaginalis*, *Candida *spp. was associated with HPV16 infection (OR = 0.10, 95% CI: 0.002–0.048). Moreover, among women with BV, vaginal *Candida* spp.colonization was associated with HPV18 infections (Mantel and Haenszel OR = 0.030, 95% CI: 0.011–0.083). When both vaginal *Candida *spp. and *T. vaginalis* were removed from the model, BV was still significantly associated with HR-HPV in all the FSWs. In contrast, when BV was removed from the model, vaginal *Candida* spp.colonization was not associated with HR-HPV, except for HPV18 (OR = 0.448, 95% CI: 0.232–0.866). The presence of vaginal *Candida* spp.colonization alone was not significantly associated with all the HR-HPV genotypes, except for HPV18 (OR = 0.448, 95% CI: 0.232–0.866). However, when *Candida* spp. colonization was present concurrently with BV or trichomoniasis, it negatively affected the outcomes for certain HPV types.

Three covariables were introduced into the multivariate logistical regression model. The distribution of BV in women with HR-HPV was statistically significant in HPV16 and HPV16-related genotypes (HPV31, 33, 35, and 52). Thus, we expect that, among the 16 HR-HPV-negative participants, 88% did not have BV. This equates to 66% of HPV31-negative, 82% of HPV33-negative, 75% of HPV35-negative, and 72% of HPV52-negative participants. In contrast, the presence of *T. vaginalis *was statistically significant in women with HPV18, HPV45, HPV66, or HPV68; the presence of *Candida *spp. was found to be statistically significant only when HPV18 was detected. This result was further associated with both *Candida *spp. and *T. vaginalis* (Table 3).

## 4. Discussion

According to Cheng et al. and Muñoz et al., HPV types may be organized according to (1) HPV16-related types (31, 33, 35, 52, and 58) or HPV types belonging to the alpha-papillomavirus-9 species, (2) HPV18-related types (39, 45, 59, 68) or HPV types belonging to the alpha-papillomavirus-7 species, or (3) others, including HPV51 and HPV66. We have used this classification system in our study [1, 13–16]. BV is the main cause of abnormal vaginal discharge in women of reproductive age. It is characterized by replacement of lactobacilli by a mixture of anaerobic and aerobic bacteria (common agents include *Gardnerella vaginalis, Mycoplasma hominis, *and* Ureaplasma urealyticum*) [17]. In women without BV, hydrogen peroxide-producing lactobacilli dominate vaginal microbiota [18]. The microorganisms responsible for BV increase the levels of mucin-degrading enzymes, which may play a role in the degradation of the gel layer that coats the vaginal and cervical epithelium and endocervical mucus [19, 20]. BV-associated microorganisms also produce cytokines and inflammatory mediators, which have been linked to pregnancy complications and may increase susceptibility to STIs [20]. Livengood et al. reported that 84% of BV-positive women also had positive results for sialidases [21]. Such enzymes may promote virulence by destroying the protective mucosal barrier and may hence increase susceptibility to cervical HR-HPV infection by facilitating adherence, invasion, and, eventually, incorporation of HPV-activated oncogenes into the genome of cells in the transformation zone. Abnormal vaginal microbiota may also be implicated in the maintenance of subclinical HR-HPV infection. Enzymes that are produced by anaerobic bacteria and are involved in the pathogenesis of BV can potentially alter immune signals and promote degradation of protective host factors, rendering women more susceptible to acquiring HR-HPV [22–25]. However, it is still not known whether BV and cervical HR-HPV infections are related because there is a biological symbiotic relationship between them or because both occur frequently in sexually active women. The role of sexual transmission in causing or promoting BV continues to be a topic of debate, as studies have shown that lesbians have a high prevalence of BV [26].

*Candida* spp. infection is the most prevalent mycosis in the majority of cases of asymptomatic colonization and vulvovaginal candidiasis. However, certain species of *Candida* are more pathogenic than the other species and induce hyphal and pseudohyphal formation and enhance proteolytic activity and antigen modulation [27]. These features enable *Candida* spp. to penetrate the mucosal surface and induce mucosal swelling, erythema, and cell exfoliation. The role of *T. vaginalis* and *Candida *spp. in HR-HPV pathogenesis has been evaluated by some authors, including Engberts et al. [28] who established that the presence of *Candida *spp. is not associated with an increased risk of acquiring HR-HPV.

In a study involving cytological analysis of samples obtained from 17,391 outpatients during January 1997–August 1998 in Brazil, 390 samples (2.24%) had alterations that were consistent with HPV infection and were sometimes associated with CIN I. The results showed that *G. vaginalis* was the most frequent bacterial agent found in women with HR-HPV infection (23.6% versus 17.4%; *P* < 0.05), while in the control group the most frequent agent was *Candida* spp. (23.9% versus 13.8%; *P* < 0.001) [29]. This result is in accordance with the findings of Wang and Lin [30] and Chakrabarti et al. [31], who also reported that there was no obvious association between the presence of *Candida* spp. and cytologic changes in the vagina. *Candida* spp. is not a cofactor in the presence of HR-HPV. However, it has been found that *Candida* spp. is more frequently found in women without cervical intraepithelial neoplasia (CIN) [32]. As reported by Watts et al. [33], *T. vaginalis* and vaginal Candida spp. colonizations were not associated with HR-HPV infection in HIV-negative women.

In contrast to the finding for BV and *T. vaginalis*, it appears that the presence of *Candida *spp. in the vagina is not a cofactor in the development of cervical cancer. This is consistent with the hypothesis that the local cervicovaginal milieu plays a role in susceptibility to HR-HPV infection since women who carry *Candida* spp. are likely to possess a healthy *Lactobacillus*-predominated vaginal microbiota, in contrast to women with dysbacteriosis [34]. Only a limited number of studies have recognized an association between the presence of *T. vaginalis* and CIN or an association between *T. vaginalis *and HR-HPV infection [34]. These findings may be explained by common etiological factors and, in particular, by the number of sex partners, which is naturally difficult to prove with a high degree of certainty [34]. Several hypotheses have been proposed to explain the pathogenesis and virulence of *T. vaginalis.* One hypothesis is that the parasite can act as a potential catalyst in the development of secondary infections, including HR-HPV, by producing a wide array of enzymes such as cysteine proteinase enzymes that are linked with cytotoxicity and the degradation of basement membrane components. Furthermore, some studies have shown that the double-stranded RNA of *T. vaginalis* is also associated with differential expression of enzymes and may therefore affect virulence [35–39]. Therefore, it is also possible that *T. vaginalis *could alter the natural history of various sexually transmitted diseases and, in particular, HR-HPV, by increasing virulence. This study clearly shows that women with positive cytological results for *T. vaginalis* are at significant risk of acquiring HR-HPV infection [40, 41].

In addition to the conventional STIs caused by *Neisseria gonorrhoeae*, *Chlamydia trachomatis*, and *T*. *vaginalis*, HR-HPV infection has been found to be common (36%), and oncogenic genotypes have been found in 44% of HR-HPV-positive patients [42].

 In another study, *T. vaginalis* was found in 80 of the patients (0.2%) [43]. Of these 80 patients, 57 were available for HR-HPV testing (age: 8 patients <30 years, 42 patients 30–50 years, and 7 patients 50 years). As controls, high-risk HR-HPV was tested for in 150 patients with normal cytological features, from each of these 3 age categories. Women with *T. vaginalis* had a significantly higher prevalence of HR-HPV than women with normal results for cervical smear tests, irrespective of age (*P* < 0.01). Noël stated that these data suggest a potential association between these 2 infectious agents because of sexual intercourse and probably because of biochemical or immunological reasons [44]. Depuydt et al. concluded that the prevalence of *T. vaginalis* in Flanders is low, with only 3.7/1,000 women infected. HR-HPV is present in 15% of these women and the peak of its prevalence occurs 20 years earlier than that of detection of *T. vaginalis*. The present study design does not allow for a definitive answer for this paradoxical increase in *T. vaginalis* prevalence in the absence of HR-HPV in the age group of 40–50 years [44].

In our study, BV and *T. vaginalis* infection was common, with oncogenic genotypes (HPV18 and HPV18-related types 59 and 68) being present in 61%, 45.5%, and 69% of positive samples. Moreover, *T. vaginalis* was present in 86% of HPV66-positive samples. These findings emphasize the importance of a cervical cancer prevention strategy, such as a cervical cytology screening program for HR-HPV, which is to be introduced soon.

## 5. Conclusions

This study suggests a positive association between BV and HR-HPVs (HPV 16, 31, 33, 35, and 52). Presence of *T. vaginalis* was statistically significant, in women with HPV 18, 45, 66, and 68. *Candida *spp. were not significantly associated with HR-HPV; they were more frequently among HR-HPV-negative women. These data confirm that screening for genital infections may reveal the simultaneous presence of different sexually transmitted microorganisms. These results suggest and emphasize the value of screening for genital infections in HR-HPV-positive patients in order to decrease the presence of other microorganisms and to reduce the probable synergistic effects of the vaginal microbiota. Prevention is important not only to avoid other STIs and their sequelae but also to reduce the influence of concomitant infection with other microorganisms on HR-HPV infection.

## Figures and Tables

**Table 1:** Genotypes in human papillomavirus positive women.

Prevalence of HPV								
HPV 16-related types (31, 33, 35, 52, and 58)			HPV 18-related types (39, 45, 59, 68)			HPV 51 Y 66		
Type of HPV	Number of sex workers	%	Type of HPV	Number of sex workers	%	Type of HPV	Number of sex workers	%
HPV 16	81 sex workers	38.9%	HPV 18	48 sex workers	23.1%	HPV 51	52 sex workers	25%
HPV 31	59 sex workers	28.4%	HPV 39	45 sex workers	21.6%	HPV 66	38 sex workers	18.3%
HPV 33	52 sex workers	25%	HPV 45	38 sex workers	18.3%			
HPV 35	39 sex workers	18.8%	HPV 56	50 sex workers	24%			
HPV 52	68 sex workers	32.7%	HPV 59	23 sex workers	11.1%			
HPV 58	54 sex workers	26%	HPV 68	31 sex workers	14.9%			

^∗^According to Muñoz et al. [12] and Li et al. [6]. 12 types were classified as high risk. 2 types as probably high risk.

**Table 2:** Characteristics of studied group: uivariate analysis.

	Bacterial vaginosis		*Trichomonas vaginalis*		*Candida* spp.	
	OR	95% confidence interval	OR	95% confidence interval	OR	95% confidence interval
HPV 16	0.058	0.023–0.147	0.083	0.033–0.213	1.051	0.600–1.840
HPV 18	0.078	0.036–0.171	0.034	0.013–0.085	0.448	0.232–0.866
HPV 31	0.209	0.103–0.425	0.199	0.0093–0.423	1.292	0.701–2.381
HPV 33	0.157	0.075–0.326	0.207	0.097–0.443	1.169	0.620–2.203
HPV 35	0.122	0.056–0.266	0.113	0.050–0.253	1.232	0.608–2.496
HPV 39	0.071	0.032–0.158	0.026	0.010–0.067	0.831	0.424–1.628
HPV 45	0.246	0.122–0.542	0.210	0.093–0.474	0.644	0.311–1.337
HPV 51	0.461	0.224–0.950	0.376	0.176–0.803	1.169	0.620–2.203
HPV 52	0.168	0.082–0.347	0.169	0.078–0.367	1.166	0.650–2.090
HPV 56	0.428	0.207–0.886	0.350	0.163–0.750	1.186	0.624–2.256
HPV 58	0.256	0.126–0.522	0.224	0.105–0.476	1.573	0.832–2.971
HPV 59	0.262	0.105–0.658	0.197	0.077–0.504	0.599	0.250–1.436
HPV 66	0.022	0.008–0.058	0.005	0.001–0.018	1.023	0.504–2.074
HPV 68	0.012	0.003–0.045	0.021	0.007–0.060	1.001	0.465–2.156

We have demonstrated a stronger association between bacterial vaginosis versus *Trichomonas vaginales* and high-risk human papillomavirus types.

**Table 3:** Multivariate analysis: odds ratio for prevalent high-risk human papillomavirus (HR-PV) among all FSWs.

	Bacterial vaginosis		*Trichomonas vaginalis*		*Candida* spp.	
	OR	95% confidence interval	OR	95% confidence interval	OR	95% confidence interval
HPV 16	0.117	0.038–0.359	*—*	*—*	*—*	*—*
HPV 18	*—*	*—*	0.047	0.012–0.177	3.423	1.3227–8.829
HPV 31	0.342	0.125–0.939	*—*	*—*	*—*	*—*
HPV 33	0.179	0.062–0.512	*—*	*—*	*—*	*—*
HPV 35	0.244	0.080–0.740	*—*	*—*	*—*	*—*
HPV 39	*—*	*—*	*—*	*—*	*—*	*—*
HPV 45	*—*	*—*	0.257	0.077–0.858	*—*	*—*
HPV 51	*—*	*—*	*—*	*—*	*—*	*—*
HPV 52	0.281	0.104–0.763	*—*	*—*	*—*	*—*
HPV 56	*—*	*—*	*—*	*—*	*—*	*—*
HPV 58	*—*	*—*	*—*	*—*	*—*	*—*
HPV 59	*—*	*—*	*—*	*—*	*—*	*—*
HPV 66	*—*	*—*	0.040	0.011–0.148	*—*	*—*
HPV 68	*—*	*—*	4.968	1.353–18.250	*—*	*—*

We detect a significant effect of BV versus trichomoniasis on incident HR-HPV.
